# Factors Associated With Dropout of Participants in an App-Based Child Injury Prevention Study: Secondary Data Analysis of a Cluster Randomized Controlled Trial

**DOI:** 10.2196/21636

**Published:** 2021-01-29

**Authors:** Jie Li, Peishan Ning, Peixia Cheng, David C Schwebel, Yang Yang, Xiang Wei, Jieyi He, Wanhui Wang, Ruotong Li, Guoqing Hu

**Affiliations:** 1 Department of Epidemiology and Health Statistics Xiangya School of Public Health Central South University Changsha China; 2 Department of Psychology University of Alabama at Birmingham Birmingham, AL United States; 3 Department of Biostatistics College of Public Health and Health Professions, Emerging Pathogen Institute University of Florida Gainesville, FL United States

**Keywords:** app-based intervention, unintentional injury, attrition, influencing factors

## Abstract

**Background:**

Mobile health (mHealth) interventions offer great potential to reach large populations and improve public health. However, high attrition rates threaten evaluation and implementation of mHealth intervention studies.

**Objective:**

We explored factors associated with attrition of study participants in an mHealth randomized controlled trial (RCT) evaluating an intervention to reduce unintentional child injury risk in China.

**Methods:**

The cluster RCT compared two groups of an app-based intervention for caregivers of 3-6–year-old children (Bao Hu San). The intervention group received unintentional child injury and parenting education, whereas only parenting education was implemented in the control group. The trial included 2920 study participants in Changsha, China, and lasted 6 months. Data on participant engagement (using the app) were collected electronically throughout the 6-month period. Associations between participant attrition and demographic characteristics, and between attrition and intervention engagement were tested and quantified separately for the intervention and control groups using the adjusted odds ratio (aOR) based on generalized linear mixed models.

**Results:**

In total, 2920 caregivers from 20 eligible preschools participated, with 1510 in the intervention group and 1410 in the control group. The 6-month attrition rate differed significantly between the two groups (*P*<.001), at 28.9% (437/1510) in the intervention group and 35.7% (503/1410) in the control group. For the intervention group, the only significant predictor of attrition risk was participants who learned fewer knowledge segments (aOR 2.69, 95% CI 1.19-6.09). For the control group, significant predictors of attrition risk were lower monthly login frequency (aOR 1.48, 95% CI 1.00-2.18), learning fewer knowledge segments (aOR 1.70, 95% CI 1.02-2.81), and shorter learning durations during app engagement (aOR 2.39, 95% CI 1.11-5.15). Demographic characteristics were unrelated to attrition.

**Conclusions:**

Engagement in the app intervention was associated with participant attrition. Researchers and practitioners should consider how to best engage participants in app-based interventions to reduce attrition.

**Trial Registration:**

Chinese Clinical Trial Registry ChiCTR-IOR-17010438; http://www.chictr.org.cn/showproj.aspx?proj=17376

**International Registered Report Identifier (IRRID):**

RR2-10.1186/s12889-018-5790-1

## Introduction

Owing to recognized advantages such as cost-effective dissemination, real-time data collection and feedback, reduced burden, flexible customization, self-monitoring capacity, and visually attractive multimedia presentation [1-3], mobile health (mHealth) technology has become increasingly popular in health intervention research and practice over the past decade. A wide range of mHealth interventions have been developed to prevent diseases and injuries, increase the compliance to recommended health interventions, and offer remote access to health services [4-10].

Despite these advantages, mHealth interventions suffer from the challenge of high dropout attrition rates compared to studies adopting traditional interventions. For example, a recent 6-month large intervention study for smoking cessation reported an attrition rate of 57% in the intervention group using an app and of only 52% in a control group using a self-help booklet as usual education [11]. Several app-based intervention studies reported high attrition rates, with estimates ranging from 38% to 84% [12-16].

When attrition is high, occurs unequally between intervention and control groups, or occurs in a nonrandom way, it threatens the validity of evaluation studies [17,18]. When dropout attrition is high in public health practice, the efficacy of an intervention is lower than desired. Therefore, researchers must prioritize exploration and understanding of the factors associated with dropout attrition of study participants. Increased understanding would lead to feasible approaches to prevent and reduce dropout attrition.

Previous research examining predictors of dropout attrition from app-based intervention studies in reducing alcohol intake [19], improving health-related behavior [20], and weight loss [21] focused primarily on the demographic characteristics of individuals who are more likely to fail to complete follow-up surveys and therefore are not retained in the research study. For example, in an evaluation of the efficacy of an app-based child burn prevention program, Burgess et al [22] reported a higher proportion of university degree holders among participants who remained in the study (28.7%, 70/244) than those who were lost to follow up (16.5%, 42/254). Despite the value of this line of research, it is equally important to explore relationships between attrition and other indicators such as engagement with the app-based intervention. Such analyses may offer new clues to reducing attrition in app-based intervention studies.

Therefore, the aim of this study was to examine associations both between attrition and demographic characteristics and between attrition and participants’ engagement in the intervention. We hypothesized that a more active and attractive type of intervention would result in greater engagement and thus a lower attrition rate in the intervention arm compared with the control arm. Replicating Burgess et al [22] and others, we also hypothesized that greater education might be related to higher retention in the intervention study. We used data collected from a cluster randomized controlled trial (RCT) examining an app intervention to prevent unintentional child injury in China to test our hypotheses.

## Methods

### Design

This research comprises secondary analyses of data from a published single-blinded cluster RCT assessing the effectiveness of an app-based intervention for caregivers to prevent unintentional injury among Chinese preschoolers [8]. The trial recruited 2920 caregivers of 3-6–year-old children from 20 eligible preschools using cluster random sampling. The randomization was performed at the school level and was stratified by type of preschool (public vs private), yielding five public and five private schools in both the intervention group (implementing unintentional child injury and parenting education) and the control group (implementing parenting education only).

Both groups engaged in a 6-month intervention delivered through a smartphone app. The intervention group received a more active and attractive intervention than the control group. Specifically, the control group received essays, games, comics, and videos twice a week, and users conducted thematic discussion activity once a month to learn about common children’s diseases and to practice parenting skills outside unintentional injury prevention. In contrast, the intervention group received all content that the control group received, but the participants were also exposed to additional similar essays, games, comics, and videos twice a week, and engaged in a second set of thematic discussions once a month to learn about parenting skills related to unintentional child injury prevention. The intervention group also had access to an app-based portal that supported communication between users and professionals concerning injury prevention knowledge and skills.

Data were collected through three caregiver surveys that were completed at baseline in December 2017 and then two follow-up surveys in March and June 2018, which corresponded to the 3rd and 6th month after the initiation of the interventions in both groups. To encourage adherence in using the app, reminders about newly released knowledge segments appeared on the scroll screen when users opened the app. Users who did not read the content in a timely manner were reminded again. We also provided financial incentives to participants after completing study of the knowledge segments and asked the preschool teachers to remind caregivers regularly to use the app. In addition, an automatic message to remind participants about using the app was sent if the user failed to log in for 1 month. To encourage compliance in completing the study surveys, we provided financial incentives and sent two automatic reminder messages to users who did not complete the follow-up surveys 3 days after they were released.

**Figure 1:**
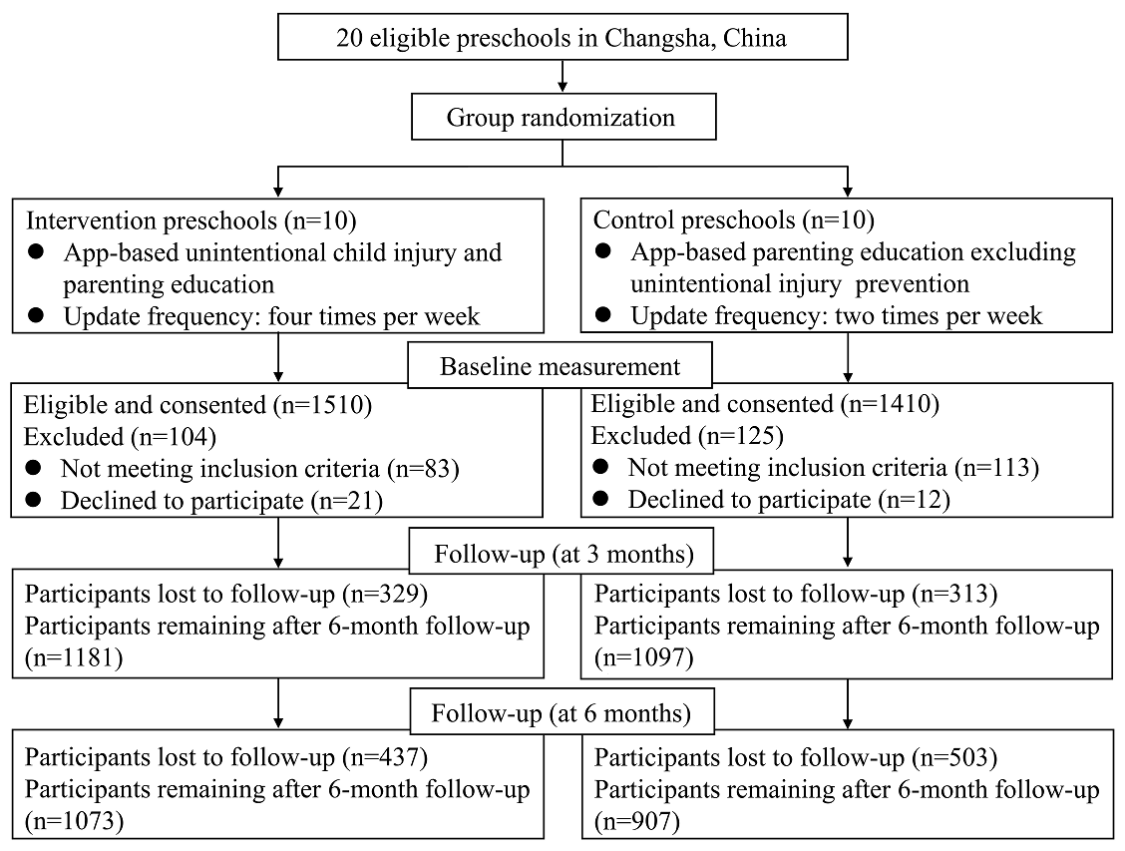
Flow diagram of the app-based intervention study.

Figure 1 illustrates the study design. The protocol was approved by the Ethics Committee of Xiangya School of Public Health, Central South University, Changsha, China (approval number: XYGW-2017-02). Full details of the RCT evaluation are published elsewhere [23].

### Outcome Measure

Attrition from the study was defined as a study participant who failed to complete both follow-up surveys at the 3rd and 6th months after initiation of the interventions.

### Independent Variables

Based on our study hypotheses and previous studies [17,22], we considered demographic factors and engagement with the app-based intervention as potential factors that would predict dropout attrition of study participants. We also considered previous training experience on unintentional injury prevention, as we hypothesized that participants might engage in the intervention or study only if they valued parenting education and if they were receiving new information that they had not learned in the past.

Data on demographic characteristics and previous training experience were obtained via a baseline survey. Caregiver demographic factors included sex, age, level of education, and monthly household income per capita. Caregiver’s age was divided into three approximately equally sized groups (less than 31 years, 31 to 34 years, and 35 years and above). Level of education was classified into three categories: junior high school and below, high school (including technical secondary school), and junior college and above. Monthly household income per capita was divided into two levels: below and above 3500 yuan (approximately US $540), according to the average income of Changsha residents in 2015 [24].

Data on engagement with the intervention were collected using the app’s backend system across full implementation of the trial; that is, user engagement information was collected in an automated fashion when users logged into the app. Available data included the webpages on the app that were visited, and on what occasions, how frequently, and for how long. This kind of information offers insight into engagement by participants based on how much time they spend with the app, and with what app features.

For this study, we collected app engagement data using four indicators: monthly login frequency, single login duration, knowledge segments learned per login, and single learning duration. Monthly login frequency was defined as the average number of times each participant used the app per month before quitting the study. Quitting was defined as the date of the last login recorded in the app backend system. Single login duration was the average online duration between opening the app and exiting it in the same session. Knowledge segments learned per login was the average number of short written statements with pictures, cartoon episodes, video recommendations, and interactive games learned in each login. Finally, single learning duration was defined as the average time spent on reading and learning individual knowledge segments during each login. To facilitate interpretation of the results, we divided all participants into three equal groups for each indicator according to the percentiles of *P*_33.4_ and *P_66.7_* (<*P*_33.4_, *P*_33.4_ to *P*_66.7_, and >*P*_66.7_).

### Statistical Analysis

Attribution rates and 95% CIs were estimated based on binomial distributions. The *χ*^2^ test was used to examine the difference in attrition rates between the intervention and control education groups. Differences in app intervention engagement measures between the two groups were evaluated using the Kruskal-Wallis *H* test. Generalized linear mixed models were used to test the associations between attrition and all independent variables. The intraclass correlation coefficient was calculated to quantify the clustering of study participants at the preschool level. Adjusted odds ratios (aORs) were calculated to quantify the size of associations after adjusting for other independent variables. All analyses were performed using SAS 9.2 (SAS Institute). Statistical significance was based on two-sided tests at the level of .05.

## Results

### Sample Characteristics

In total, 2920 caregivers from 20 eligible preschools in Changsha, China participated, with 1510 caregivers assigned to the intervention group and 1410 assigned to the control group (Table 1). Participants in the intervention group were slightly more likely to be male, to have a higher monthly household income per capita, and to have received injury prevention education in the past 3 months. There also were differences in engagement: participants in the intervention group were more likely to engage in the website based on all four metrics than those in the control group (*P*<.001).

**Table 1:** Demographic and engagement characteristics of study participants.

Characteristic					Intervention (n=1510)		Control (n=1410)		*P* value
Age (years), mean (SD)					32.7 (5.0)		33.1 (5.7)		.06
**Sex, n (%)**									
	Male			490 (32.5)		364 (25.8)			<.001
	Female			1020 (67.5)		1046 (74.2)			
**Educational level, n (%)**									
	Junior high school and below			1017 (67.3)		951 (67.5)		.98	
	High school			386 (25.6)		357 (25.3)			
	College and above			107 (7.1)		102 (7.2)			
**Household income per capita per month (US $), n (%)**									
	<540			367 (24.3)		272 (19.3)		.001	
	≥540			1143 (75.7)		1138 (80.7)			
**Injury prevention education in past 3 months, n (%)**									
	Yes			918 (60.8)		708 (50.2)		<.001	
	No			592 (39.2)		702 (49.8)			
Monthly login frequency, median (IQR)				3.9 (5.5)		3.0 (5.5)		<.001	
Single login duration (seconds), median (IQR)				204.9 (221.8)		176.8 (198.7)		<.001	
Knowledge segments learned per login, median (IQR)				0.7 (1.7)		0.5 (1.2)		<.001	
Single learning duration (seconds), median (IQR)				61.9 (162.8)		44.9 (131.0)		<.001	

### Engagement Indicators Between the Intervention and Control Groups

The intervention group had significantly higher engagement than the control group based on all four engagement indicators. This was true both for participants who completed the study and for those who failed to complete the study (Table 2).

**Table 2:** Differences in app engagement indicators between the intervention and control groups within 6 months.

Variable	Completers (n=1980)			Lost to follow up (n=940)		
	Intervention, median (IQR)	Control, median (IQR)	*P* value	Intervention, median (IQR)	Control, median (IQR)	*P* value
Monthly login frequency (N)	3.9 (6.0)	3.5 (6.0)	.03	3.9 (4.6)	2.0 (4.0)	<.001
Single login duration (seconds)	222.2 (232.6)	195.0 (214.2)	<.001	156.3 (174.2)	142.6 (192.9)	<.001
Knowledge segments learned per login (N)	1.0 (1.7)	0.8 (1.3)	<.001	0.2 (0.9)	0.1 (0.7)	.02
Single learning duration (seconds)	86.0 (172.2)	72.7 (163.7)	.01	16.0 (82.8)	7.4 (54.3)	.009

### Attrition Rate

The 6-month attrition rate for the intervention group was 28.9% (437/1510; 95% CI 26.6%-31.2%), whereas that for the control group was 35.7% (503/1410; 95% CI: 33.2%-38.2%). The attrition was significantly higher in the control group than in the intervention group (OR 1.36, 95% CI 1.17-1.59) (Multimedia Appendix 1).

### Influencing Factors

Given the distinct attrition rates across the two groups, we performed multivariable analysis separately for each group. As shown in Table 3, the attrition rates of caregivers in the intervention group with “sometimes” monthly login frequency was higher than that of those with “often” monthly login frequency (*P*=.03). The attrition rates of caregivers with short and average single login durations were significantly higher than those for caregivers with long single login durations (*P*<.001 and *P*=.03, respectively). The attrition rates of caregivers with few and average number of knowledge segments learned per login were significantly higher than that for caregivers completing many knowledge segments learned per login (*P*<.001 and *P*=.003, respectively). Caregivers with short and average single learning durations had higher attrition rates than those with a long single learning duration (*P*<.001 and *P*=.002, respectively). The multivariable analysis showed that caregivers who learned fewer knowledge segments per login had a substantially higher attrition risk than those who learned more knowledge segments per login after adjusting for sex, age group, level of education, monthly household income per capita, receiving injury prevention education in the past 3 months or not, and the three other intervention engagement measures (Table 3).

As shown in Table 4, the attrition rate of caregivers in the control group with “seldom” monthly login frequency was higher than that for those with “often” monthly login frequency (*P*=.003). The attrition rates of caregivers with short and average single login durations were higher than that of caregivers with long single login durations (*P*<.001 and *P*=.009, respectively). The attrition rates of caregivers with few and average knowledge segments learned per login were much higher than that for caregivers with many knowledge segments learned per login (*P*<.001 and *P*=.001, respectively). The attrition rates of caregivers with short and average single learning durations were higher than that for caregivers with long single learning durations (*P*<.001 and *P*=.002, respectively). The multivariable analysis for attrition yielded three significant predictors of attrition. Caregivers with a lower monthly login frequency, who learned fewer knowledge segments per login, and who had shorter single learning durations had significantly higher attrition rates than those using the app more often per month (seldom vs often), learning more knowledge segments per login (average vs many), and having a longer single learning duration (short vs long) (Table 4).

**Table 3:** Six-month attrition rates of caregivers of preschoolers in the intervention group (N=1510).

Variable			Attrition rate (%) (95% CI)	OR^a^ (95% CI)	aOR^b^ (95% CI)
Total^c^			28.9 (26.6-31.2)	N/A^d^	N/A
**Sex**					
	Male		24.3 (20.5-28.1)	Reference	Reference
	Female		31.2 (28.4-34.0)	1.14 (0.85-1.54)	1.24 (0.90-1.70)
**Age group (years)**					
	<31		34.6 (30.4-38.8)	1.43 (1.00-2.05)	1.32 (0.90-1.94)
	31-34		28.4 (24.8-32.0)	1.32 (0.92-1.91)	1.36 (0.93-1.99)
	≥35		23.0 (19.0-27.0)	Reference	Reference
**Level of education**					
	Junior high school and below		40.2 (30.9-49.5)	1.28 (0.76-2.17)	1.23 (0.69-2.22)
	High school		32.4 (27.7-37.1)	0.97 (0.70-1.35)	0.98 (0.68-1.40)
	College and above		26.5 (23.8-29.2)	Reference	Reference
**Monthly household income per capita (US $)**					
	<540		31.9 (27.1-36.7)	0.96 (0.70-1.33)	0.90 (0.63-1.30)
	≥540		28.0 (25.4-30.6)	Reference	Reference
**Received injury prevention education in the past 3 months**					
	Yes		26.3 (23.5-29.1)	Reference	Reference
	No		33.1 (29.3-36.9)	1.20 (0.90-1.60)	1.09 (0.80-1.48)
**Monthly login frequency^e^**					
	Seldom (<*P*_33.4_)		31.7 (27.4-36.0)	1.24 (0.88-1.74)	0.88 (0.60-1.29)
	Sometimes (*P*_33.4_-*P*_66.7_)		30.5 (26.6-34.4)	1.46 (1.05-2.01)	1.26 (0.89-1.78)
	Often (>*P*_66.7_)		25.2 (21.6-28.8)	Reference	Reference
**Single login duration^e^**					
	Short (<*P*_33.4_)		40.4 (35.8-45.0)	2.68 (1.91-3.77)	1.26 (0.83-1.90)
	Average (*P*_33.4_-*P*_66.7_)		27.9 (24.0-31.8)	1.49 (1.06-2.09)	1.01 (0.69-1.48)
	Long (>*P*_66.7_)		20.7 (17.3-24.1)	Reference	Reference
**Knowledge segments learned per login^e^**					
	Few (<*P*_33.4_)		48.9 (44.3-53.5)	4.76 (3.34-6.77)	2.69 (1.19-6.09)
	Average (*P*_33.4_-*P*_66.7_)		25.9 (22.0-29.8)	1.86 (1.30-2.66)	1.38 (0.83-2.30)
	Many (>*P*_66.7_)		15.9 (12.9-18.9)	Reference	Reference
**Single learning duration^e^**					
	Short (<*P*_33.4_)		48.1 (43.5-52.7)	4.67 (3.26-6.67)	1.76 (0.78-3.95)
	Average (*P*_33.4_-*P*_66.7_)		26.1 (22.3-29.9)	1.92 (1.34-2.75)	1.42 (0.88-2.31)
	Long (>*P*_66.7_)		15.9 (12.9-18.9)	Reference	Reference

^a^OR: odds ratio.

^b^aOR: adjusted odds ratio.

^c^The intraclass correlation coefficient was 0.15 for level two (preschool). Tests for multicollinearity indicated a low level of multicollinearity (tolerance>0.10 and variance inflation factor<5 for all predictors). The Hosmer-Lemeshow goodness-of-fit suggested that overall model fit was acceptable (*P*=.75).

^d^N/A: not applicable.

^e^These variables were equally divided into three groups based on the *P*_33.4_ and *P*_66.7_ percentiles.

**Table 4:** Six-month attrition rates of caregivers of preschoolers in the control group (N=1410).

Variable			Attrition rate (%) (95% CI)	OR^a^ (95% CI)	aOR^b^ (95% CI)	
Total^c^			35.7 (33.2-38.2)	N/A^d^	N/A	
**Sex**						
	Male		33.2 (28.4-38.0)	Reference	Reference	
	Female		36.5 (33.6-39.4)	1.04 (0.76-1.42)	1.05 (0.76-1.47)	
**Age group (years)**						
	<31		38.4 (33.7-43.1)	1.03 (0.72-1.48)	0.99 (0.68-1.45)	
	31-34		38.0 (34.0-42.0)	1.25 (0.90-1.74)	1.15 (0.81-1.63)	
	≥35		30.2 (25.9-34.5)	Reference	Reference	
**Education level**						
	Junior high school and below		47.1 (37.4-56.8)	1.13 (0.68-1.87)	1.02 (0.58-1.80)	
	High school		40.1 (35.0-45.2)	0.94 (0.69-1.30)	0.88 (0.63-1.24)	
	College and above		32.8 (29.8-35.8)	Reference	Reference	
**Monthly household income per capita (US $)**						
	<540		38.6 (32.8-44.4)	1.02 (0.73-1.42)	1.04 (0.71-1.51)	
	≥540		35.0 (32.2-37.8)	Reference	Reference	
**Received injury prevention education in past 3 months**						
	Yes		30.6 (27.2-34.0)	Reference	Reference	
	No		40.7 (37.1-44.3)	1.20 (0.91-1.58)	1.19 (0.89-1.59)	
**Monthly login frequency^e^**						
	Seldom (<*P*_33.4_)		46.2 (42.0-50.4)	1.77 (1.27-2.48)	1.48 (1.00-2.18)	
	Sometimes (*P*_33.4_-*P*_66.7_)		30.5 (26.1-34.9)	1.03 (0.72-1.48)	1.10 (0.75-1.61)	
	Often (>*P*_66.7_)		27.4 (23.2-31.6)	Reference	Reference	
**Single login duration^e^**						
	Short (<*P*_33.4_)		44.7 (40.5-48.9)	2.15 (1.53-3.02)	0.98 (0.65-1.48)	
	Average (*P*_33.4_-*P*_66.7_)		34.3 (30.0-38.6)	1.68 (1.18-2.39)	1.21 (0.82-1.80)	
	Long (>*P*_66.7_)		25.9 (21.7-30.1)	Reference	Reference	
**Knowledge segments learned per login^e^**						
	Few (<*P*_33.4_)		53.6 (49.3-57.9)	4.43 (3.04-6.46)	1.92 (0.88-4.20)	
	Average (*P*_33.4_-*P*_66.7_)		31.5 (27.4-35.6)	2.15 (1.46-3.15)	1.70 (1.02-2.81)	
	Many (>*P*_66.7_)		17.5 (13.7-21.3)	Reference	Reference	
**Single learning duration^e^**						
	Short (<*P*_33.4_)		54.2 (49.9-58.5)	4.29 (2.98-6.19)	2.39 (1.11-5.15)	
	Average (*P*_33.4_-*P*_66.7_)		30.4 (26.3-34.5)	1.98 (1.36-2.89)	1.45 (0.89-2.35)	
	Long (>*P*_66.7_)		18.5 (14.8-22.2)	Reference	Reference	

^a^OR: odds ratio.

^b^aOR: adjusted odds ratio.

^c^The intraclass correlation coefficient was 0.15 for level two (preschool); tests for multicollinearity indicated a low level of multicollinearity (tolerance>0.10 and variance inflation factor<5 for all predictors); the Hosmer-Lemeshow goodness-of-fit suggested that overall model fit was acceptable (*P*=.85).

^d^N/A: not applicable.

^e^These variables were equally divided into three groups based on the *P*_33.4_ and *P*_66.7_ percentiles.

## Discussion

### Principal Findings

The 6-month attrition rate of study participants was 28.9% in the intervention group and was 35.7% in the control group. As hypothesized, higher attrition risks were associated with low app intervention engagement in both the intervention and control groups after controlling for demographic variables and previous training experience on unintentional injury prevention. Compared to the intervention group, the control group had a higher attrition rate, which was primarily explained by lower app intervention engagement. Demographic factors seemed to not be associated with attrition in either group.

### Interpretation of Findings

Attrition in our study was quite similar to that reported at 6-month follow up for an educational app on car seat use [25] (34.3%, 387/1129), but was significantly lower than the rate of 51% (254/498) reported in an app-based study of scald burn prevention [22]. A wide range of factors likely explain participant attrition from engagement in an app-based intervention, including design of the app, assessment measures for participant compliance, individual characteristics of participants, and cultural differences across countries.

Previous studies [13,22,26] generally report that female, younger, and less educated individuals have a higher likelihood of attrition. We could not confirm these findings in either the univariable or the multivariable analyses. One possible explanation for this result is the fact that we conducted our study in China, a middle-income country with a collectivist culture that differs from other countries and regions where the previous studies were conducted, such as the United States and Australia. Another possible explanation is related to our multivariable findings, which indicate that participant engagement was a strong predictor of participant attrition after controlling for participant demographics. In both groups, engagement in the intervention was significantly associated with attrition. These results support the hypothesis that a high proportion of participants lost to follow up in app-based interventions may simply be a result of nonusage of the app [17]. If participants perceive the app as helpful and beneficial, they continue to use it and then engage in research surveys.

We found that the attrition rate in the intervention group was lower than that in the control group, a finding we attribute to the less engaging aspects of the parenting education app for the control group. Specifically, the control group received new material twice a week and thematic discussions once a month, while the intervention group received new material four times a week and engaged in thematic discussions twice a month. The intervention group also had access to experts to ask questions and receive consultation, a feature not provided to the control group. These data concord with the findings of Kelders et al [27] who reviewed 101 web-based intervention studies and found that increased interaction with a counselor, more frequent intended usage, more frequent updates, and more extensive employment of dialogue support better adherence.

### Implications

Our findings have two major implications. First, they emphasize the unignorable impact of high dropout attrition in mHealth intervention studies. High attrition must be evaluated strictly before drawing conclusions about the efficacy of an intervention, and research findings should be interpreted cautiously when attrition rates are high, vary substantially across comparison groups, or when missing values (dropouts) occur in a nonrandom fashion. Second, the results underscore the need for careful design and implementation of mHealth interventions. The comparatively high attrition rate among participants with less frequent and engaging exposure to educational materials highlights the need for mHealth interventions to be designed and implemented with appealing features. They must attract users and increase engagement in the intervention.

### Study Limitations

This study was primarily limited by the RCT design. We do not have data concerning engagement or attrition rate after 6 months since the study ended at that point. Further, other potentially relevant factors for dropout were not available for analysis, including participant preferences, attitudes, and views about the app-based interventions, all of which may influence attrition [28]. These limitations should be considered in future research.

### Conclusions

Dropout attrition was high in an RCT examining app-based interventions for unintentional child injury and parenting education versus only parenting education among caregivers of Chinese preschoolers. Engagement in the interventions differed across the two groups, and attrition was significantly associated with intervention engagement in both groups. Greater engagement with the app led to lower attrition. Attrition must be considered by researchers, policymakers, and practitioners when evaluating and implementing mHealth interventions. Efforts to engage users are critical to reduce attrition. Future research might consider feasible strategies to improve and maintain compliance of study participants to mHealth intervention programs.

## Appendix

Multimedia Appendix 1 Six-month attrition rates of caregivers of preschoolers (N=2920).

## Abbreviations

aOR: adjusted odds ratio
mHealth: mobile health
RCT: randomized controlled trial
