# Supplementary material for: Factors Associated With Dropout of Participants in an App-Based Child Injury Prevention Study: Secondary Data Analysis of a Cluster Randomized Controlled Trial
Source: J Med Internet Res. 2021 Jan 29;23(1):e21636. doi: 10.2196/21636 (PMC7880806; doi:10.2196/21636)
Supplement: Multimedia Appendix 1 [file jmir_v23i1e21636_app1.docx]

**Table MA1. Six-month attrition rates of caregivers of preschoolers (N=2920).**

| Variable | | Attrition rate (%) (95% CI) | OR^a^ (95% CI) | aOR^b^ (95% CI) |
| --- | --- | --- | --- | --- |
| **Group**^c^ | |  |  |  |
|  | Intervention group | 28.9 (26.6-31.2) | Reference | Reference |
|  | Control group | 35.7 (32.2-38.2) | 1.36 (1.17-1.59) | 1.19 (1.00-1.40) |
| **Sex** | |  |  |  |
|  | Male | 28.1 (25.1-31.1) | Reference | Reference |
|  | Female | 33.9 (31.8-35.9) | 1.09 (0.89-1.33) | 1.15 (0.93-1.43) |
| **Age group** | |  |  |  |
|  | <31 years | 36.3 (33.1-39.4) | 1.22 (0.96-1.55) | 1.14 (0.89-1.46) |
|  | 31-34 years | 33.1 (30.4-35.8) | 1.29 (1.02-1.62) | 1.22 (0.96-1.55) |
|  | ≥35 years | 26.7 (23.7-29.6) | Reference | Reference |
| **Level of education** | |  |  |  |
|  | Junior high school and below | 43.5 (36.8-50.3) | 1.20 (0.85-1.69) | 1.11 (0.76-1.62) |
|  | High school | 36.1 (32.6-39.5) | 0.96 (0.77-1.19) | 0.92 (0.73-1.16) |
|  | College and above | 29.5 (27.5-31.5) | Reference | Reference |
| **Monthly household income per capita** | | | |  |
|  | <3500 Chinese Yuan | 34.7 (31.0-38.4) | 0.99 (0.79-1.23) | 0.99 (0.78-1.27) |
|  | ≥3500 Chinese Yuan | 31.5 (29.6-33.4) | Reference | Reference |
| **Received injury prevention education in the past 3 months** | | | | |
|  | Yes | 28.2 (26.0-30.4) | Reference | Reference |
|  | No | 37.3 (34.6-39.9) | 1.20 (1.00-1.44) | 1.14 (0.94-1.38) |
| **Monthly login frequency** | |  |  |  |
|  | Seldom (<*P*_33.4_) | 39.7 (36.7-42.8) | 1.52 (1.21-1.90) | 1.14 (0.88-1.46) |
|  | Sometimes (*P*_33.4_-*P*_66.7_) | 30.5 (27.6-33.5) | 1.24 (0.99-1.56) | 1.17 (0.92-1.49) |
|  | Often (>*P*_66.7_) | 26.2 (23.4-28.9) | Reference | Reference |
| **Single login duration** | |  |  |  |
|  | Short (<*P*_33.4_) | 42.7 (39.6-45.8) | 2.38 (1.90-2.99) | 1.12 (0.85-1.46) |
|  | Average (*P*_33.4_-*P*_66.7_) | 30.9 (28.0-33.8) | 1.58 (1.26-1.99) | 1.10 (0.86-1.42) |
|  | Long (>*P*_66.7_) | 22.9 (20.3-25.6) | Reference | Reference |
| **Knowledge segments learned per login** | | |  |  |
|  | Few (<*P*_33.4_) | 51.4 (48.2-54.6) | 4.57 (3.59-5.81) | 2.27 (1.34-3.83) |
|  | Average (*P*_33.4_-*P*_66.7_) | 28.8 (26.0-31.6) | 2.00 (1.56-2.55) | 1.51 (1.09-2.11) |
|  | Many (>*P*_66.7_) | 16.5 (14.2-18.9) | Reference | Reference |
| **Single learning duration** | |  |  |  |
|  | Short (<*P*_33.4_) | 51.4 (48.2-54.5) | 4.47 (3.52-5.68) | 2.05 (1.22-3.44) |
|  | Average (*P*_33.4_-*P*_66.7_) | 28.2 (25.4-31.0) | 1.95 (1.53-2.49) | 1.45 (1.05-2.00) |
|  | Long (>*P*_66.7_) | 17.0 (14.6-19.3) | Reference | Reference |

^a^OR: odds ratio.

^b^aOR: adjusted odds ratio.

^c^The intraclass correlation coefficient (ICC) was 0.15 for level two (preschool). Tests for multicollinearity indicated a low level of multicollinearity (tolerance > 0.10 and variance inflation factor < 5 for all predictors). The Hosmer-Lemeshow goodness-of-fit suggested that overall model fit was acceptable (*P* = 0.99).
